# Intercomparison of Two Fluorescent Dyes to Visualize Parasitic Fungi (Chytridiomycota) on Phytoplankton

**DOI:** 10.1007/s00248-021-01893-7

**Published:** 2021-12-02

**Authors:** Isabell Klawonn, Susanne Dunker, Maiko Kagami, Hans-Peter Grossart, Silke Van den Wyngaert

**Affiliations:** 1 Department of Experimental Limnology, Leibniz-Institute of Freshwater Ecology and Inland Fisheries (IGB), 12587 Berlin, Germany; 2 Leibniz Institute for Baltic Sea Research (IOW), Seestrasse 15, 18119 Rostock, Germany; 3 Department for Physiological Diversity, Helmholtz Centre for Environmental Research (UFZ), 04318 Leipzig, Germany; 4 German Centre for Integrative Biodiversity Research (iDiv), 04103 Leipzig, Germany; 5 Faculty of Science, Toho University, Funabashi, Chiba 274-8510 Japan; 6 Faculty of Environment and Information Sciences, Yokohama National University, Yokohama, Kanagawa 240-8502 Japan; 7 Institute of Biochemistry and Biology, Potsdam University, 14476 Potsdam, Germany; 8 WasserCluster Lunz, Biologische Station, Dr. Carl Kupelwieser Promenade 5, 3293 Lunz am See, Austria; 9 Department of Biology, University of Turku, Vesilinnantie 5, 20014 Turku, Finland

**Keywords:** Calcofluor White, Wheat germ agglutinin, Epifluorescence microscopy, Imaging flow cytometry, Microbial pathosystems

## Abstract

Fungal microparasites (here chytrids) are widely distributed and yet, they are often overlooked in aquatic environments. To facilitate the detection of microparasites, we revisited the applicability of two fungal cell wall markers, Calcofluor White (CFW) and wheat germ agglutinin (WGA), for the direct visualization of chytrid infections on phytoplankton in laboratory-maintained isolates and field-sampled communities. Using a comprehensive set of chytrid–phytoplankton model pathosystems, we verified the staining pattern on diverse morphological structures of chytrids via fluorescence microscopy. Empty sporangia were stained most effectively, followed by encysted zoospores and im-/mature sporangia, while the staining success was more variable for rhizoids, stalks, and resting spores. In a few instances, the staining was unsuccessful (mostly with WGA), presumably due to insufficient cell fixation, gelatinous cell coatings, and multilayered cell walls. CFW and WGA staining could be done in Utermöhl chambers or on polycarbonate filters, but CFW staining on filters seemed less advisable due to high background fluorescence. To visualize chytrids, 1 µg dye mL^−1^ was sufficient (but 5 µg mL^−1^ are recommended). Using a dual CFW–WGA staining protocol, we detected multiple, mostly undescribed chytrids in two natural systems (freshwater and coastal), while falsely positive or negative stained cells were well detectable. As a proof-of-concept, we moreover conducted imaging flow cytometry, as a potential high-throughput technology for quantifying chytrid infections. Our guidelines and recommendations are expected to facilitate the detection of chytrid epidemics and to unveil their ecological and economical imprint in natural and engineered aquatic systems.

**Supplementary Information:**

The online version contains supplementary material available at 10.1007/s00248-021-01893-7.

## Introduction

Parasitism—as a strong selective pressure in nature [1]—is one of the most dominating microbial interactions in plankton communities [2, 3]. Yet, microparasites are rarely considered in trophic interactions and biogeochemical processes in the aquatic environment, partly due to limitations in observing them in mixed microbial communities. Phytoplankton, which contributes to almost half of the primary production on Earth [4], is susceptible to various parasites [5]. For instance, members of the fungal division Chytridiomycota, referred to as chytrids, infect all major phytoplankton groups [6, 7], thereby altering the composition of phytoplankton communities [8–10], with cascading effects on microbial interactions and the flow of energy through aquatic food webs—a concept referred to as the fungal shunt [11] and mycoloop [12]. Moreover, chytrid epidemics may suppress the development of phytoplankton blooms [8, 13, 14], potentially limiting the spread of harmful algae blooms [15] but also causing severe problems for algae mass culturing during feedstock or biofuel production [16, 17]. Chytrid epidemics thus profoundly affect ecosystem functioning with ecological and economic implications [6, 18, 19].

Since the pioneering work on chytrids in British lakes [20], chytrids have been primarily studied locally in a few lakes and on a limited number of isolates [6], mostly because their small and inconspicuous thalli are frequently overlooked or misidentified by the untrained eye when using light microscopy. During the last decade, advances in DNA sequencing assays have substantially broadened the observed diversity and distribution of chytrids in aquatic habitats, reaching from high-altitude lakes [21], coastal regions [22, 23] to the deep-sea [24, 25] across various climate zones [7, 26–31]. This recently discovered diversity and biogeography, however, remains to be complemented with direct observations of chytrid abundances and host identities in most of those habitats.

Chitin-binding dyes, as markers for fungal cell walls, in combination with fluorescence microscopy are often used to determine infection prevalences, i.e., the proportion of host cells that is infected [32]. More recently, quantitative PCR (qPCR) [33], a chitin-binding probe [34], and fluorescence *in situ* hybridization (FISH) [35, 36] have been applied to detect chytrids. However, these three approaches have crucial limitations: qPCR has only been tested on free-swimming zoospores, so far, but not on host-associated sporangia [33], the previously used chitin-binding probe is not commercially available any longer, and FISH represents a rather expensive, low-throughput method. The use of chitin-binding dyes and fluorescence microscopy, therefore, remains the simplest and most widely used method to directly visualize chytrid–phytoplankton associations. Chitin-binding dyes include Calcofluor White (CFW), wheat germ agglutinin (WGA), Congo Red, Lactophenol-cotton blue, and Trypan Blue [37]. Of those, CFW has been used most frequently [38], while WGA is the least toxic one. We thus focused on CFW and WGA, which both have been applied on pelagic [39] and benthic communities [37]. The fluorescent dye CFW binds nonspecifically to beta-1,3 and 1,4-linked polysaccharides which include chitin but also cellulose, the latter being present in cell walls of some phytoplankton taxa and fungi-like organisms, which can obscure chytrid detections [39]. The fluorescently-tagged lectin WGA instead binds specifically to N-acetylglucosamine, i.e., the monomeric unit of the polymer chitin. Nevertheless, despite its specific chitin-binding properties, comparatively few studies have used WGA for chytrid staining [e.g., 40, 41].

To clarify the applicability of CFW and WGA in chytrid staining assays, we conducted a rigorous intercomparison of CFW vs. WGA staining on nine isolated pathosystems and field-sampled plankton communities. We were able to validate the staining of various morphological features of isolated chytrids during their life cycle. We further quantified the effect of different dye concentrations and sample storage times on the staining quality and verified the performance of CFW and WGA staining in combination with two widely used fixatives (Lugol and paraformaldehyde, PFA). Moreover, we demonstrate the application of WGA staining in combination with imaging flow cytometry as a potential set-up for high throughput quantification of chytrid infections. Finally, we offer guidelines for a CFW–WGA dual staining protocol, which we applied on natural plankton communities, to improve the detection of chytrid epidemics in diverse artificial and natural aquatic ecosystems.

## Materials and Methods

Specifications and recipes of all chemical solutions, and a step-by-step staining protocol including epifluorescence microscopy are detailed in the supplementary information (Supplementary Text S1 and S2).

### Staining Various Morphological Features of Chytrids in Multiple Model Systems

We grew nine taxonomically different host–chytrid pathosystems (Table 1) in batch cultures, as previously described (see Supplementary Text S3). Sub-samples of each co-culture were preserved with Lugol (alkaline, 10 µL mL^−1^) in 2-mL tubes and stored at 4 °C in darkness. Prior microscopy, samples were destained from Lugol by adding sodium thiosulfate (Na_2_S_2_O_3_, final conc. 7.6 mM) [42] and thereafter dual stained with CFW and WGA (Fluorescent Brightener 28 and WGA-Alexa Fluor™ 488 Conjugate) for 15 min in the dark (5 µg mL^−1^ of each dye). The dual staining was justified since no competitive staining was observed herein and earlier [39, 41], allowing for a direct comparison of both dyes in the same sample. Stained chytrids were evaluated in Utermöhl chambers on the same day under an inverted fluorescence microscope (Nikon Eclipse Ti2, used throughout this study and therefore referred to as microscope in the following) at 600 × magnification using two fluorescence channels (WGA: 482/35 nm excitation/536/40 nm emission, CFW: 377/50 excitation/415 LP emission).

**Table 1:** Model pathosystems used for comparative CFW and WGA staining, including their isolation date and location. References (Ref.) include the chytrid descriptions (if available). Genbank accession numbers are listed in the Supplementary (Table S3)

Host-chytrid system	Taxa	ID	Date	Location	Ref.
*Asterionella formosa - Rhizophydiales *sp. #1	Diatom	AST-A1	Nov-2016	Lake Stechlin (GER)	-
	Rhizophydiales	AST-CHY1	Dec-2016	Lake Stechlin (GER)	
*Ulnaria *sp.* - Zygophlyctis planktonica*	Diatom	HS-SYN2	Apr-2015	Haussee (GER)	[43]
	Zygophlyctidiales	SVdW-SYN-CHY1	Feb-2016	Melzersee (GER)	
*Staurastrum *sp.* - Chytridiales *sp.	Desmid	STAU-ULLS3	Sep-2017	Ullswater (GBR)	-
	Chytridiales	STAU-CHY RBA3	Oct-2017	Rimov reservoir (CZE)	
*Staurodesmus - Rhizophydiales *sp. #2	Desmid	STAU-ULLS2	Sep-2017	Ullswater (GBR)	-
	Rhizophydiales	STAU-CHY6	Sep-2017	Dormant water (GBR)	
*Staurastrum *sp.* -Rhizophydiales *sp. #3	Desmid	STAU-ULLS3	Sep-2017	Ullswater (GBR)	-
	Rhizophydiales	STAU-CHY RBA5	Oct-2017	Rimov reservoir (CZE)	
*Staurastrum *sp.* - Staurastromyces oculus*	Desmid	STAU1	Oct-2014	Lake Stechlin (GER)	[44]
	Rhizophydiales	STAU-CHY3	Jul-2015	Lake Stechlin (GER)	
*Eudorina elegans - Algomyces stechlinensis*	Green algae	PAN1	Oct-2014	Lake Stechlin (GER)	[45]
	Lobulomycetales	SVdW- EUD3	Dec-2015	Lake Stechlin (GER)	
*Yamagishiella unicocca - Dangeardia mamillata*	Green algae	PAN4	Oct-2014	Lake Stechlin (GER)	[45]
	Incertae sedis	SVdW- EUD2	Jul-2015	Lake Stechlin (GER)	
*Yamagishiella unicocca - Endocoenobium eudorinae*	Green algae	PAN4	Oct-2014	Lake Stechlin (GER)	[45]
	Polyphagales	SVdW- EUD1	Jun-2015	Lake Stechlin (GER)	

**Figure. 1:**
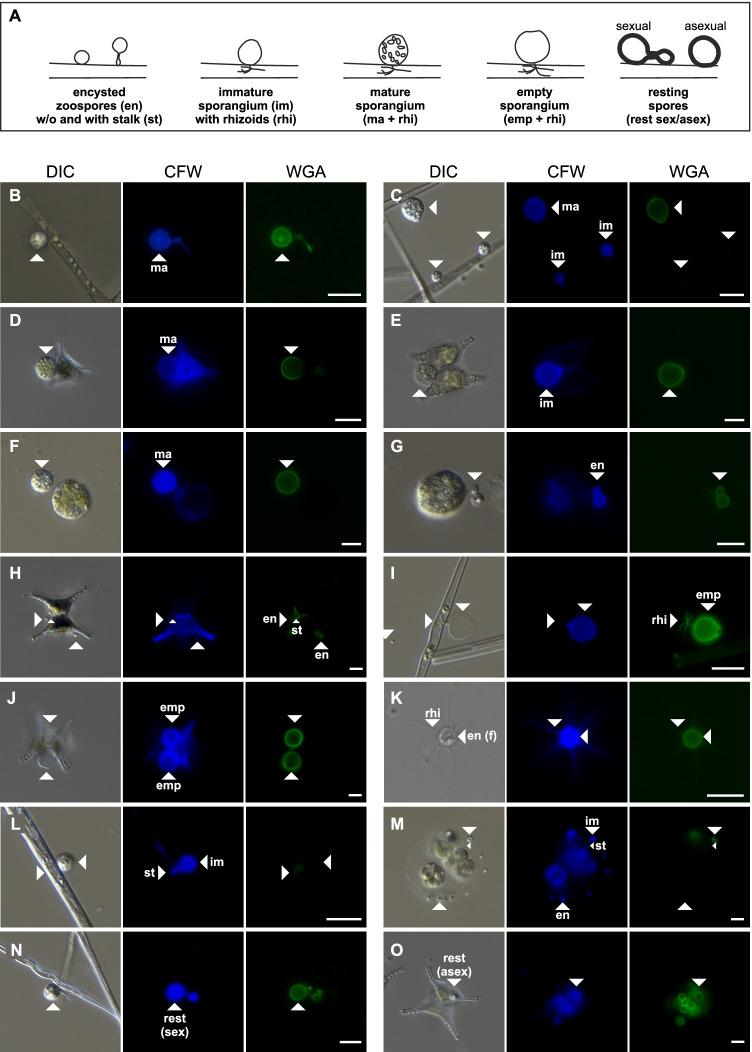
**(A)** Scheme of various morphological features during the life cycle of parasitic chytrids. Infections emerge as free-living, motile zoospores settle and encyst onto a phytoplankton cell [46]. After encystment, the parasite penetrates and expands into the host’s interior via hyphae-like rhizoids, through which nutrients are conveyed from the host to the parasite. The host-derived nutrients nurture the exterior structure to a mature sporangium, which produces and releases new zoospores into the ambient water upon maturation, leaving behind an empty sporangium. To overcome periods of low host abundances, many chytrids can produce resting spores, sexually [46] or asexually [44]. **(B–O)** Microscopy images of various phytoplankton‒chytrid model systems used for the comparative CFW and WGA staining (data shown in Fig. 2). **B** *Asterionella–Rhizophydiales* #1, **C, I, L, N** *Ulnaria–Zygophlyctis*, **D, H, J** *Staurastrum–Chytridiales*, **E** *Staurastrum–Staurastromyces*, **F, M** *Eudorina–Algomyces*, **G** *Yamagishiella–Endocoenobium*, and **O** *Staurastrum–Rhizophydiales*. **K** shows the rather uncommon zoospores of *Endocoenobium*, which develop immotile spores with a cell wall and rhizoid-like structures for host attachment. White scale bars are 10 µm. Abbreviations: DIC differential interference contrast. CFW Calcofluor White, WGA wheat germ agglutinin, en—encysted zoospores (host-associated), en (f)—encysted zoospores (freely suspended, not host-associated), im/ma—immature/mature sporangia, emp—empty sporangia, rest (sex/asex)—resting spores (sexual/asexual), rhi—rhizoids, st—stalks

The life cycle of chytrids involves various development stages and morphological features, which we addressed by validating the staining patterns on those multiple features (Fig. 1A). Features included (1) encysted zoospores (i.e., initially attached zoospores), (2) immature sporangia (larger than encysted zoospores but not yet matured, no visible zoospores), (3) mature sporangia (zoosporangia with visible zoospores inside), (4) empty sporangia (cell wall remains of the sporangia after zoospore discharge), (5) resting spores (resting stages with thickened cell walls), (6) rhizoids (rhizoidal structures inside the host cell), and (7) stalks (extensions of the sporangia to physically attach to the host’s cell wall). In addition, we examined encysted, immotile, freely suspended zoospores [i.e., not attached to any host cell, in contrast to (1)] which were only present in the *Yamagishiella*–*Endocoenobium* system. Of each morphological feature, 25 events (in 15 out of 96 instances 5–20 events) were evaluated in parallel with both fluorescence channels. The staining patterns were qualitatively evaluated and assigned to three categories: (1) entirely stained, i.e., the entire morphological feature displayed a bright, homogeneous fluorescent signal, 2) partly stained, i.e., the morphological feature did not display a homogeneous fluorescent signal or the fluorescence was weaker compared to other morphological structures of the same species, and 3) not stained, if no fluorescent signal was detectable. Additionally, we evaluated the staining patterns on live cells (no Lugol-preservation) in three selected systems (*Asterionella–Rhizophydiales*, *Ulnaria–Zygophlyctis*, and *Staurastrum–Staurastromyces*).

### Dye Concentrations, Storage Times, and Sample Preservation/Preparation Types

Using the *Asterionella‒Rhizophydiales* #1 co-culture, we validated the effect of different dye concentrations and storage times on the enumeration of chytrid and host cells, and their emitted fluorescence intensity. Three different CFW and WGA concentrations (1, 5, and 25 µg mL^−1^, storage overnight) and storage times (1 day, 4 weeks, and 6 months, dye concentration was 5 µg mL^−1^) were tested. Since infection prevalences are commonly determined by enumerating encysted zoospores and sporangia [14], we verified the staining of those structures, if not indicated differently.

Samples were either preserved with Lugol (alkaline, 10 µL mL^−1^) and inspected in Utermöhl chambers or preserved with paraformaldehyde (PFA, final conc. 1.5%, fixation overnight) and inspected on polycarbonate filters (PC, 0.2 µm, 25 mm)—hereafter Lugol- and PFA-preserved samples, respectively (see Supplementary Figure S1 for a schematic workflow). In this way, we minimized the exposure of the applicant to toxic volatile PFA during sample preparation and microscopy. In addition, we stained live cells (no preservation, day 0), applying the same staining protocol as for Lugol-preserved samples but without Lugol addition. Lugol-preserved samples were stored in 2-mL tubes at 4 °C, while PFA-preserved samples were stored on filters at -20 °C. Prior microscopy, Lugol-preserved samples were destained from Lugol and stained with CFW and WGA, as described above but with separate CFW and WGA staining (no dual staining). PFA-preserved cells were stained in liquid and thereafter filtered onto PC filters if cells were analyzed immediately; or, if stored at -20 °C, cells were filtered first and thereafter stained on the filter. In the latter case, the filters were submerged in the dye solution for 15 min in darkness and washed twice with 1 mL CHU-10 medium and 1 mL MilliQ to remove excess dye, followed by air-drying.

Chytrid sporangia and *Asterionella* cells were counted in triplicates under the microscope at 300 × magnification, using three fluorescence channels (CFW: 387/11 excitation/442/46 emission, WGA: 482/35 nm excitation/536/40 nm emission, and chlorophyll autofluorescence: 635/18 nm excitation/680/42 nm emission). In total, we counted ~ 300–1,200 *Asterionella* cells (non-infected and infected) and ~ 50–500 encystments/sporangia per replicate (*n* = 3–4 chambers or filters). Since single *Asterionella* cells could carry multiple infections, we report the ratio of total host cells to chytrid infections (the latter defined as the sum of encysted zoospores and im-/mature sporangia) instead of reporting the infection prevalence (which does not account for multiple infections on single host cells). CFW-stained filters displayed a high background fluorescence, which precluded the determination of meaningful sporangia counts. CFW-stained sporangia were consequently not analyzed on filters (but in Utermöhl chambers).

After counting, we imaged the fluorescent signal of WGA-stained sporangia and chlorophyll-autofluorescent *Asterionella* cells. CFW-stained sporangia were also imaged but the CFW images turned out to be overexposed, and thus, they were not used for any further data processing. We took 20 z-stack images (521 × 521 µm, 0.4 µm increments over 10–15 µm depth, Nikon DS-Ri2 camera, 16 MP) per replicate (*n* = 3–4 chambers or filters), each image covering several *Asterionella* colonies with and without infections. Images were always taken with the same settings (e.g., light intensity, gain, and exposure time), except that the autofluorescent signal of Lugol-preserved *Asterionella* cells was amplified through a higher gain (20.9x) as compared to PFA-preserved cells (1.8 × gain). This higher gain was chosen since the autofluorescence of Lugol-preserved cells was otherwise poorly visible. Fluorescence intensities of WGA-stained sporangia and autofluorescent *Asterionella* cells were analyzed on the mono-color images from the WGA and chlorophyll autofluorescence channels, respectively. Each z-stack was merged to a composite image with one focal plane (NIS-Elements AR software, v. 5.01.00, function: extended depth of focus) and further processed in ImageJ (v.1.51p) [47]. In ImageJ, color images were converted into 16-bit images, followed by thresholding to separate bright foreground pixels (fluorescent sporangia or *Asterionella* cells) from dark background pixels. The fluorescence intensity in the hereby separated cells was analyzed as mean grey values via particle analysis (function: analyze particles). Only chytrid cells and *Asterionella* cells emitted a fluorescence above the threshold on the WGA and chlorophyll-autofluorescent images, respectively, and thus only those cells were included in the final data set.

### Imaging Flow Cytometry of WGA-Stained Sporangia

A sub-sample of the WGA-stained *Asterionella‒Rhizophydiales* co-culture (no fixative, 5 µg WGA mL^−1^) was pre-filtered through a 100 µm cell strainer, to avoid clogging the tubes of the flow cytometer (*Asterionella* colonies were mostly < 100 µm). The filtrate was split into two—one for flow cytometry and one for microscopy analyses. Flow cytometry was conducted with an imaging flow cytometer (ImageStream®X MK II, Luminex Corporation, US) equipped with three lasers (488 nm/200 mW, 561 nm/200 mW, and 785 nm/80 mW) and two CCD-cameras [48, 49]. Measurements were performed with laser intensities of 488 nm/1 mW, 561 nm/40 mW, and 785 nm/0.5 mW [the intensity of the 561 nm laser was reduced by a neutral density-filter (optical density 1.0), and 40 mW refers to the original laser intensity]. Dulbecco`s phosphate-buffered saline without calcium and magnesium (Biowest, Nuaillé, France) was used as a sheath-fluid. For each triplicate, ~ 50 µL were analyzed automatically at 200 × magnification (120 × 512 µm field of view, numeric aperture 0.5, pixel size 1 × 1 µm). Data acquisition with the INSPIRE software (v. 201.1.0.693) was finished when 1,000 events (including *Asterionella* cells and associated sporangia but excluding speed calibration beads) were measured. Infections were counted based on the images for WGA fluorescence (488 nm excitation, 528/65 nm emission), brightfield, and autofluorescence (488 nm excitation, 702/85 nm emission) using the IDEAS software (v. 6.2.187.0). As a direct comparison, *Asterionella* cells and associated sporangia were counted in Utermöhl chambers (triplicates) via microscopy, as described above. CFW-stained sporangia were not analyzed via imaging flow cytometry since the instrument was not equipped with a UV laser, as this laser has a shorter lifetime and is more expensive than the ones used herein.

### CFW and WGA Staining of Field-Sampled Communities

Mixed plankton communities were sampled with a plankton net (HydroBios, 25 µm mesh size) from surface waters (0–5 m) in a mesotrophic lake (Lake Stechlin, 53°08′34.6"N and 13°01′41.9"E) and a coastal station in the Baltic Sea (54°08.76'N and 11°50,58'E, salinity 8–20‰, both Northern Germany) in 2018, 2020 and 2021, fixed with Lugol and stored at 4 °C. Before microscopy, samples were dual-stained (5 µg dye mL^−1^, after destaining from Lugol), as described above, and inspected in Utermöhl chambers at 200–400 × magnification. We were able to detect 1–156 sporangia per phytoplankton taxon, which were evaluated in parallel for their positive WGA and CFW-staining. A step-by-step protocol for the dual-staining method, and statistical analyses are included in the supplementary information (Text S2 and S4).

## Results

### Effectivity of CFW and WGA Staining for Various Morphological Features of Chytrids

**Fig. 2:**
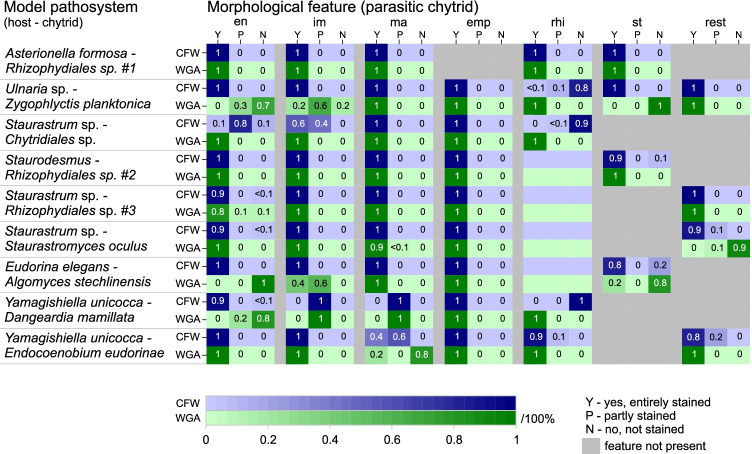
Effectivity of CFW and WGA staining for various morphological features of chytrids indiverse phytoplankton‒chytrid model systems. The color scale and detailed numbers indicate the fraction of entirely/partly stained or unstained features. In four pathosystems, rhizoids were presumably present but not visible after staining (no numbers given). Grey boxes indicate features that could not be tested for their staining patterns since they were not developed by the chytrids in our samples. Empty sporangia of *Rhizophydiales* on *Asterionella* dissolved after zoospore discharge and could thus not be evaluated. Abbreviations are defined in the caption of Fig. 1

Morphological features of chytrids in the model systems showed a similar CFW and WGA staining pattern after dual-staining, with some exceptions (Figs. 1 and 2). Encysted zoospores and sporangia were visible to ≥ 90% with both dyes in most systems. As an exception, encysted zoospores and im-/mature sporangia in the *Ulnaria*‒*Zygophlyctis, Yamagishiella‒Dangeardia, and Yamagishiella‒Endocoenobium* systems were stained (entirely or partly) to 0‒80% with WGA, while CFW stained those features to ≥ 90% (e.g., Fig. 1C and L for *Ulnaria‒Zygophlyctis*, and 1G for *Yamagishiella‒Endocoenobium*, Fig. 2). Empty sporangia associated with post-infected host cells were stained with both dyes to 100% in all systems. Resting spores and rhizoids, if present, were mostly more effectively stained with WGA (10–100%) than with CFW (0–100%). In four out of nine model systems, stalks were part of the chytrid morphology and they were stained with CFW (80–100%) and WGA (20–100%). Free-swimming zoospores commonly do not have a chitinous cell wall and they are, therefore, not stained by chitin-binding dyes. Zoospores of *Endocoenobium*, however, encyst freely in the water and develop a special type of immotile spores with a cell wall and rhizoid-like structures through which they attach to their motile host [45]. Those immotile infectious spores were entirely or at least partly stained with CFW and WGA (Fig. 1K , data are not included in Fig. 2 since this feature was only observed in one pathosystem).

**Fig. 3:**
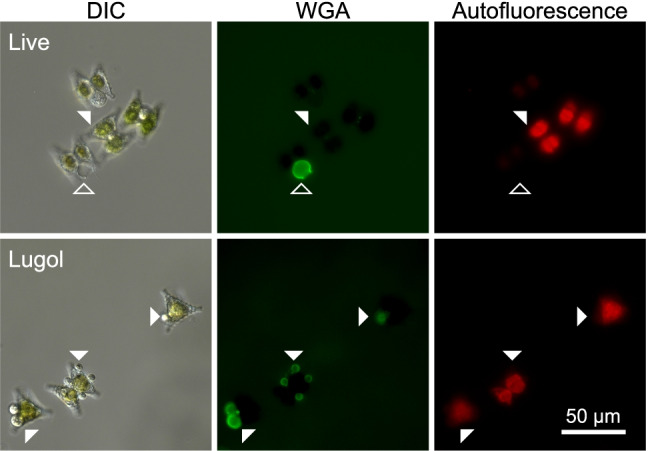
Micrographs depicting the WGA staining pattern in the *Staurastrum‒Staurastromyces* pathosystem in live (no preservation) and Lugol-preserved samples. In live samples, WGA visualized empty but not mature sporangia (non-filled and filled arrowheads, respectively). In Lugol-preserved samples, by contrast, mature sporangia were stained with WGA. See Supplementary Figure S2 for exemplary CFW images. DIC—differential interference contrast, WGA—wheat germ agglutinin

Interestingly, host-associated encysted zoospores and im-/mature sporangia were stained differently in live vs. Lugol-preserved samples in the three tested pathosystems. WGA failed to visualize those features in live samples of *Staurastrum-Staurastromyces* and *Ulnaria-Zygophlyctis*, whereas the staining was successful after Lugol preservation (Fig. 3). In the *Asterionella–Rhizophydiales* system, such a different staining pattern was not apparent since WGA visualized encysted zoospores and sporangia in both live and Lugol-preserved samples. In contrast to WGA, CFW stained encysted zoospores and sporangia in both live and Lugol-preserved samples in all three systems (Supplementary Figure S2). Empty sporangia were stained with CFW and WGA in live and Lugol-preserved samples in all three systems.

### Dye Concentrations and Storage Times, Utermöhl (Lugol) vs. Filter (PFA), and Microscopy vs. Flow Cytometry (*Asterionella‒Rhizophydiales* System)

**Fig. 4:**
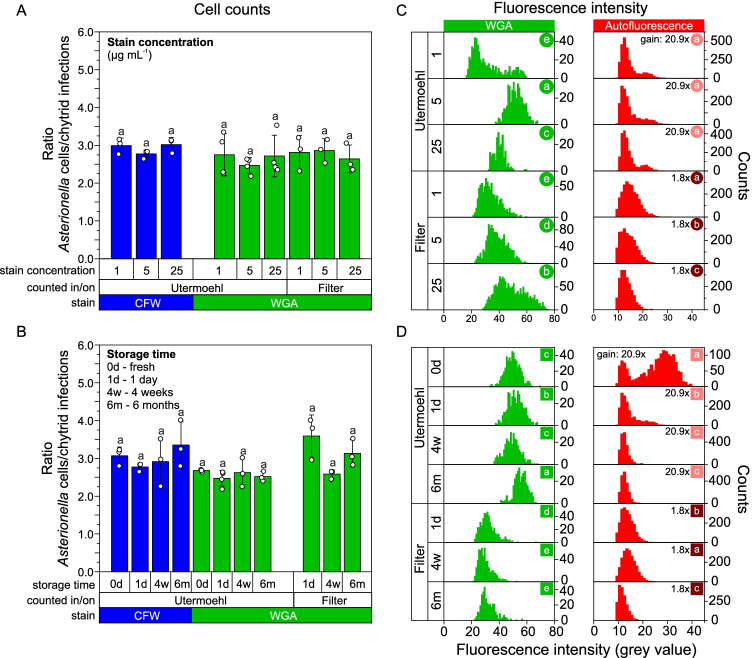
Microscopy analyses of the *Asterionella‒Rhizophydiales* pathosystem after CFW and WGA staining using different dye concentrations **(A, C)** and sample storage times **(B, D)**. **A and B** Cells were counted in Utermöhl chambers (Lugol fixation) and on polycarbonate filters (PFA fixation). Samples from day 0 represent live samples (no preservation). Shown are mean ± s.d. (*n* = 3–4, s.d. ≤ 22%). **C and D** Fluorescence intensity emitted by WGA-stained sporangia and chlorophyll-autofluorescent *Asterionella*, determined via image analyses. Statistical differences in the data distribution between different groups are indicated by different letters (a‒d, Kruskal–Wallis test, *p* > 0.05). Multiple group comparisons were run separately for WGA-stained sporangia and chlorophyll-autofluorescent *Asterionella* cells at different dye concentrations and storage times in Utermöhl chambers and on filters (indicated by the different colors and symbols). The chlorophyll autofluorescence of Lugol-fixed *Asterionella* cells was rather low compared to PFA-fixed cells, and thus, Lugol-fixed cells in Utermöhl chambers were imaged at a higher gain (20.9x) than PFA-fixed cells on filters 1.8x. Fluorescence data of CFW-stained sporangia are not shown due to overexposure (*i.e.*, cells displayed max. color values)

The mean ratio of total *Asterionella* cells to chytrid infections (i.e., the sum of encysted zoospores and im-/mature sporangia) was 2.9 ± 0.4 (mean ± s.d., *n* = 87). This ratio was independent of the dye concentration (1‒25 µg mL^−1^, *p* > 0.05, Df = 8) and storage time (1 day‒6 months, *p* > 0.05, Df = 7, Kruskal–Wallis H-test, Fig. 4A, B). The intensity of the green fluorescence of WGA-stained sporangia was rather similar across different dye concentrations and storage times (Fig. 4C, D). Intensities of the green fluorescence of WGA-stained sporangia were lower on PC filters (PFA-preserved) than in Utermöhl chambers (Lugol-preserved, *p* < 0.05, Kruskal–Wallis, Df = 4). Nonetheless, WGA-stained sporangia were well distinguishable from the background fluorescence on/in both filters and Utermöhl chambers. The diatom’s autofluorescence was highest in live samples, and if a fixative was added, it was better preserved by PFA than by Lugol.

**Fig. 5:**
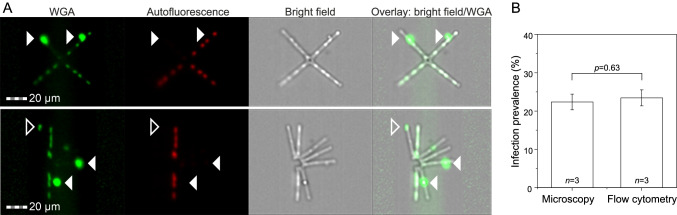
**A** Visualization of WGA-stained sporangia in the *Asterionella–Rhizophydiales* pathosystem via imaging flow cytometry. The green fluorescence (WGA) depicts mature sporangia (filled arrowhead) and one post-infected *Asterionella* cell with rhizoids (open arrowhead). **B** The obtained infection prevalence was statistically not significantly different between microscopy and flow cytometry analyses (*t*-test, *p* = 0.63, *Df* = 5)

In addition to microscopy, we successfully enumerated WGA-stained sporangia in the *Asterionella‒Rhizophydiales* model system via imaging flow cytometry. Cells were well recognizable on the bright field, autofluorescence, and WGA images (Fig. 5A). Consequently, flow cytometry and microscopy yielded similar estimates for host abundances and infection prevalences (host abundances: 125,571 ± 12,232 and 116,354 ± 12,592 cells mL^−1^, *p* = 0.50, infection prevalence: 22 ± 2 and 23 ± 2%, *p* = 0.63, Df = 5, respectively, *t*-test, Fig. 5B).

### CFW and WGA Staining of Field-Sampled Chytrid Sporangia

In field-sampled plankton communities, we observed sporangia associated with diverse phytoplankton taxa, mostly including cyanobacteria and diatoms (Table 2). As a key difference between dyes, WGA effectively illuminated chytrid sporangia that were associated with the dinoflagellates *Peridinium* and *Ceratium*. By contrast, CFW brightly stained the cellulosic thecae of those dinoflagellates, thereby preventing the visualization of the associated sporangia (Supplementary Figure S3). The dual-staining was successful in limnic and oligo-/mesohaline water.

**Table 2:** Dual-staining of chytrid sporangia that were associated with various phytoplankton taxa in natural water

Phytoplankton		No. of sporangia		Sampling date
Group	Genus	CFW	WGA	dd/mm/20yy*
Cyanobacteria	*Planktothrix*	156	146	18/04/18 (LS)
Cyanobacteria	*Pseudanabaena*	61	61	25/08/18 (LS)
Cyanobacteria	*Dolichospermum* (heterocytes)	6	6	25/08/18 (LS)
Cyanobacteria	*Dolichospermum* (vegetative cells)	6	5	25/08/18 (LS)
Cyanobacteria	*Dolichospermum* (vegetative cells)	40	40	06/07/21 (BS)
Diatoms	*Cerataulina*	40	40	01/12/20 (BS)
Diatoms	*Chaetoceros *	40	40	13/10/20 (BS)
Diatoms	*Fragilaria*	40	39	18/04/18 (LS)
Diatoms	*Fragilaria*	8	7	16/11/18 (LS)
Diatoms	*Pseudo-nitzschia*	40	40	01/12/20 (BS)
Diatoms	*Synedra*	24	24	18/04/18 (LS)
Dinoflagellates	*Peridinium*	0^†^	20	16/11/18 (LS)
Dinoflagellates	*Ceratium*	0^†^	13	25/08/18 (LS)
Desmids	*Staurastrum*	42	42	16/11/18 (LS)
Chlorophyta	*Eudorina*	12	12	25/08/18 (LS)
Chrysophyta	*Dinobryon*	1^‡^	1	25/08/18 (LS)

* LS – Lake Stechlin (freshwater), BS – Baltic Sea (coastal)

† Brightly CFW-stained thecae obscured the visualization of associated sporangia

‡ Cellulosic lorica of the host were also stained with CFW

## Discussion

Chytridiomycota appear almost pervasive in freshwater and coastal marine environments as their detection in recent DNA sequencing assays has substantially ramped up their diversity and biogeography [28, 41, 50–53]. Hence, to quantify their ecological and economical imprint, there is a need to detect chytrid–phytoplankton associations via direct observations. Our methodological discussion therefore aims to facilitate a simple, reliable, and standardized screening of chytrid infections in various microplankton communities during case studies and routine monitoring programs.

### Intercomparison of CFW and WGA Staining

The staining patterns of various morphological features in the model systems were similar yet not identical for CFW and WGA. In live samples, WGA did not stain encysted and im-/mature sporangia in the *Staurastrum–Staurastromyces* and *Ulnaria–Zygophlyctis* cultures (Fig. 3). Those phytoplankton cells and chytrid sporangia were presumably coated with a polysaccharide-rich mucilage [54, 55], which results from the secretion of polymeric substances from phytoplankton [55–59] and chytrid cells [60]. Such cell coatings may limit the accessibility of WGA to the fungal cell walls since WGA is a large macromolecule (~ 35 kDa), which poorly diffuses into gelatinous coatings (mucilage) of phytoplankton cells or higher fungi [39, 61]. After Lugol fixation, the mucilage likely dispersed, allowing for the positive WGA-staining (Fig. 3). In support of this observation, lectin binding has been shown to improve after chemical treatment (e.g., KOH or trypsin hydrolysis) which unmasks the mucilage on higher fungi (*Fusarium*) and the human placenta [61, 62], while KOH has also been used previously during CFW-based chytrid staining [63]. Similarly, WGA was shown to stain intracellular rhizoids in empty frustules of *Asterionella* but not in live cells [39], indicating that WGA poorly penetrates through intact cell walls and membranes. CFW is instead a comparably small hydrophilic molecule (~ 1 kDa) which may more easily interfuse gelatinous layers and cell structures, as indicated by the positive CFW staining in live cells (Figure S2). Sample fixation is, therefore, more crucial for WGA than for CFW staining, and we recommend fixing the samples for a couple of hours (overnight) before microscopy, to allow for an effective WGA binding to the fungal cell walls.

Rhizoids were WGA stained in five out of nine systems, while CFW stained those in only three systems (Fig. 2). In the remaining systems, the rhizoids were likely unbranched and rather short, and thus, their visualization may require a higher resolution technique than light microscopy, such as transmission electron microscopy [44]. In a few instances, encysted and im-/mature sporangia, and stalks were stained less effectively with WGA than with CFW, even after sample fixation (Fig. 2). Besides the mentioned mucilage, which may partly remain after Lugol fixation, WGA binding was potentially hampered by a multi-layered structure of the fungal cell wall. In higher fungi (*Aspergillus, Cryptococcus*, and *Saccharomyces*), cell walls can consist of two layers of which the outer layer is without chitin [64, 65], while the inner layer includes chitin, but the chitin-binding sites are presumably poorly accessible to WGA. The observed differential pattern in WGA-staining between species and even between different morphological features of the same species may thus result from cell coatings with carbohydrates, multilayered cell wall structure [60], and/or variable contents of N-acetylglucosamine in chytrid cell walls, potentially limiting the WGA-binding. Differential staining patterns in WGA vs. CFW stained cells, and live vs. fixed samples could therefore indicate differences in chytrid taxonomy and morphology.

In previous studies, a wide range of dye concentrations has been applied to visualize fungal cell walls—3‒50 µg WGA mL^−1^ [37, 39, 41, 62, 66, 67] and 25‒10,000 µg CFW mL^−1^ [37–39, 41, 66, 68]. By comparison, we tested concentrations in the lower range (1, 5, and 25 µg mL^−1^). The lowest concentration (1 µg mL^−1^), and hence the most cost-efficient option, was sufficient for both WGA and CFW-based identification and enumeration of chytrid sporangia (Fig. 4A). The appropriate dye concentration may, however, depend on the chitin concentration in the water. We thus recommend 5 µg dye mL^−1^, as chitin concentrations in natural waters are expected to be in the lower µg mL^−1^ range [69]. CFW-stained chytrids were difficult to distinguish from the background fluorescence on PC filters after staining with 1 µg CFW mL^−1^, and even indistinguishable from the background at 5 and 25 µg CFW mL^−1^. Rasconi et al. [38] reported that chytrids were still visible on filters that were stained with 35 µg CFW mL^−1^, but also mentioned that higher dye concentrations precluded any accurate assessment of sporangia.

Inspecting chytrid infections in Utermöhl chambers was beneficial for the host‒parasite recognition since (1) the contrast between fungal cell walls and the background fluorescence was more distinct in Utermöhl chambers than on filters, and (2) host cells and their associated parasites could be inspected in parallel under the bright field and fluorescence channels. In this way, also non-fluorescent structures of the phytoplankton host (e.g., silica frustules, spines, or flagella) were visible in Utermöhl chambers, whereas those morphological structures were poorly visible on filters. Moreover, Lugol is less toxic than PFA, and Utermöhl chambers are the standard tool in phytoplankton ecology and monitoring [70]. Accordingly, chytrid staining in those chambers can be readily implemented in, e.g., phytoplankton monitoring programs. The host’s autofluorescence was well preserved in PFA-fixed cells (as compared to Lugol-fixed cells), which we stored on filters, while PFA-fixation is also applicable to liquid samples and subsequent flow cytometry. Filters can furthermore be stored long-termed at -20 °C, even after microscopy, and filter samples are well compatible with bacterial enumeration and identification assays [11, 71].

### Cross-Reactivity and False Positives

CFW binds to beta-1,4 or 1,3-linked polysaccharide polymers, as present in chitin but also in cellulose, keratin, collagen, and elastin [72]. CFW thus, as a critical disadvantage, stains cellulosic cell walls of multiple phytoplankton groups, including thecate dinoflagellates and some desmids and diatoms [39, 66, 73, 74]. Likewise, in our field samples, the cellulosic theca of *Peridinium* and *Ceratium* (dinoflagellates) were brightly stained with CFW, preventing the visualization of their associated sporangia (Table 2, Supplementary Figure S3). This may explain the hitherto relatively few observations of chytrids associated with dinoflagellates, and if they were detected, dinoflagellates had lost their thecae at advanced infection stages [75–77]. Moreover, CFW binds to the cellulosic cell walls of oomycetes [37, 78], which can be misidentified as chytrids. Oomycetes and chytrids are dissimilar in their taxonomy (oomycetes are grouped into stramenopiles, together with diatoms) [79]. Yet, both resemble each other in their morphology and lifestyle since the partly globose, ovoid sporangia and free-swimming zoospores of oomycetes can parasitize phytoplankton, similar to chytrids. As a distinction, however, sporangia of oomycetes are mostly endobiotic (develop inside the host cell), in contrast to the mostly epibiotic sporangia of chytrids [80]. WGA usually does not stain oomycetes since their cell walls lack chitin, but traces of chitin [79, 81] and a rare case of WGA-staining on oomycetes have been reported [82]. Parasites belonging to the fungi(-like) lineages Cryptomycota and Aphelida may also be misidentified as chytrids. Yet, there is mounting evidence that parasitic Cryptomycota often lack chitinous cell walls [83, 84] and act as hyperparasites of chytrids rather than as parasites of phytoplankton [85], while Aphelida are mostly known as endobiotic parasites of phytoplankton [86], like oomycetes. Extracellular infective cysts of Cryptomycota and Aphelida, however, can contain chitin and show positive WGA-staining [87, 88]. We additionally detected WGA-stained choanoflagellates in association with phytoplankton cells, as shown previously [89, 90], and also a positive CFW staining of those flagellates can be expected [90]. Choanoflagellates, however, are distinct in their morphology, with their funnel-shaped collar and ovoid/spherical basal cell body (Figure S4).

WGA cross-reacts with N-acetylglucosamine residues in the peptidoglycan layer of Gram-positive bacteria [91]. Yet, those bacteria are mostly smaller (1‒2 µm) than sporangia (5‒30 µm, if matured), and their false identification can be avoided by DAPI-counterstaining, to confirm that the target cell is a eukaryote with a distinct DNA-containing nucleus [41]. WGA staining can moreover be combined with other fluorescent dyes, e.g., during CARD-FISH [11, 71], as various WGA conjugates (with different wavelengths) are commercially available. CFW emits blue light after UV excitation, and it therefore overlaps with DAPI, making DAPI co-staining not applicable, but co-staining with the alternative nucleic acid stain SYTOX green has been applied successfully [92].

During our field sampling, we used neutral and acidic Lugol for sample preservation and realized a pH effect on the staining pattern. That is, we observed loosely aggregated flocs that were stained with WGA when using acidic instead of neutral Lugol (final pH in the samples was 4 and 7, respectively, Supplementary Figure S5). This pH effect was less prominent for CFW, i.e., flocs were stained neither at acidic nor neutral pH. We thus recommend using neutral or alkaline instead of acidic Lugol for observing chytrids. Yet, neutral or alkaline Lugol preserve the silica cell walls of diatoms less well than acidic Lugol, and thus short storage times should be considered, while the preservation of coccolithophorids requires neutral/alkaline Lugol because of their calcareous coccoliths [93]. In conclusion, the staining with CFW and WGA is broadly applicable to various study designs, but stain-specific advantages and disadvantages need to be considered (summarized in Table 3).

**Table 3:** Advantages ( +) and disadvantages (–) of CFW and WGA staining for the detection of chytrids

		CFW		WGA
Binding specificity	±	non-specific fluorochrome that binds to beta-(1,4) and (1.3)-glucans, including chitin	+	chitin-binding lectin, which specifically binds to N-acetyl-D-glucosamine (in chitin) and N-acetyl-D-neuraminic (sialic) acid residues
Cross-reactivity	–	cross-reactivity with cellulose, chitosan, keratin, collagen, and elastin	–	cross-reactivity with peptidoglycan layer of Gram-positive bacteria
	–	binding affinity to cellulosic cell walls of phytoplankton can hamper a successful distinction between phytoplankton cells and associated sporangia		
	–	oomycetes with cellulosic cell walls can be misidentified as chytrids		
	–	binding to cell walls of choanoflagellates expected	–	binding to cell walls of choanoflagellates observed
Staining efficiency*	+	Sporangia: 88–100%	–	Sporangia: 0–100%
	–	Rhizoids: 0–100%	+	Rhizoids: 100%
	+	Resting spores: 100%	–	Resting spores: 8–100%
	+	Stalks: 80–100%	–	Stalks: 0–100%
Fluorescence	–	fixed wavelength (Ex_max_/Em_max_ 355/433 nm)	+	flexible wavelengths: WGA lectins are commercially available with different conjugated fluorophores (e.g., Alexa-Fluor 350, 488, 555, 647, etc., Fluorescein)
Co-staining	+	DAPI co-staining is not possible (but co-staining with SYTOX green has been applied)	+	DAPI co-staining possible, combined applications with CARD-FISH have been published
Sample preparation	+	Utermöhl chambers: good applicability	+	Utermöhl chambers: good applicability
	–	PC filters: limited applicability (high	+	PC filters: good applicability (moderate
		background already at 1 µg CFW mL^−1^)		background even at 25 µg WGA mL^−1^)
	±	Flow cytometry: not tested herein	+	Flow cytometry: good applicability
Sample fixation	+	live, non-fixed cells: successful staining of sporangia	–	live, non-fixed: partly unsuccessful staining of mature sporangia (limited accessibility to WGA binding sites)
	+	Lugol and PFA-preserved cells: successful staining of sporangia	+	Lugol and PFA-preserved cells: successful staining of sporangia
	+	acidic Lugol did not induce formation/visualization of unspecific CFW-stained flocs	–	acidic Lugol induced formation/visualization of unspecific WGA-stained flocs (neutral/alkaline Lugol preferable)
Storage	+	room temperature	–	-20 °C, repeated freeze/thawing cycles should be avoided
	+	long-term storage (> 1 year)	+	stable for at least one year (manufacturer instructions)
Costs	+	inexpensive (0.10 USD per 1 mL sample)	+	inexpensive (0.40 USD per 1 mL sample)

*Based on entirely/partly stained features as shown in Fig. 2

± indicates a neutral evaluation.

### Imaging Flow Cytometry

We validated imaging flow cytometry as a time-efficient, high throughput method to quantify chytrid–phytoplankton associations. The flow cytometer imaged 1,000 host cells within 10–15 min, whereas it took 60–90 min to count 1,300 cells under the microscope. In this way, a large number of cells could be evaluated quickly via flow cytometry, while the measuring capacity of the instrument is even higher (5,000 particles s^−1^). Both methods agreed in the obtained host cell abundances and infection prevalence, with a similar accuracy (s.d. of the mean cell abundances was ca. 10%). As a proof of concept, we successfully separated the *Asterionella* cells from chytrid sporangia on the flow cytometry images (see Supplementary Figure S6 for an image example), which could be used for automated image analyses. Such automated analyses might be particularly applicable to well-defined, homogeneous co-cultures. Their application on more heterogeneous field-sampled populations, however, is expected to require more advanced algorithms for automated cell distinction and remains to be tested. In addition to cell counting, noninfected and infected phytoplankton cells could potentially also be sampled individually via intelligent image-activated cell sorting (iIACS) [94] and thereafter used for, e.g., single-cell genome/transcriptome sequencing.

### Recommendations

Detecting chytrid infections in mixed field populations can be difficult for the untrained eye. To facilitate the screening of chytrids via microscopy, we recommend:

1. CFW–WGA dual staining after cell fixation (overnight), to more reliably identify any cross-reactivities and false positives/negatives, as compared to mono-staining (Table 3, see staining protocol in the supplementary information, Text S2,).
2. Using an inverted microscope, equipped with a bright field and a long-path filter block (e.g., excitation 377/50, emission 415 DAPI LP). Thereby, the sample can concurrently be illuminated/excited with white light and UV light in Utermöhl chambers, visualizing both the phytoplankton host and CFW-stained sporangia (no switching between filter blocks needed).
3. Searching for well-defined globose to ovoid structures (i.e., sporangia with distinct cell walls and little shape irregularity). Those sporangia and their infected host usually display no or little autofluorescence, as compared to non-infected phytoplankton cells (but note that the host’s autofluorescence fades within days after Lugol preservation while it is preserved longer by PFA).
4. The re-occurrence of sporangia on the same phytoplankton taxon can be evaluated as a positive sign of chytrid infections since chytrids are often host-specific (at the species or genus level) [85].

Using these guidelines, we detected multiple, partly undescribed chytrid–phytoplankton associations in a freshwater and coastal system (Table 2). Hence, our intercomparison and recommendations shall aid in detecting chytrid infections also in other habitats, to advance our mechanistic and quantitative understanding of the effects of chytrid epidemics on trophic interactions and element cycling in diverse aquatic environments.

## Supplementary Information

Below is the link to the electronic supplementary material.

Supplementary file1 (PDF 2654 KB)

## Data Availability

Data shown in Figure 2 and the ImageJ scripts used for image analyses (data in Figure 4 and S6) are achieved in the open-access PANGAEA database (https://www.pangaea.de/).
