# Supplementary material for: Intercomparison of Two Fluorescent Dyes to Visualize Parasitic Fungi (Chytridiomycota) on Phytoplankton
Source: Microb Ecol. 2021 Dec 2;85(1):9–23. doi: 10.1007/s00248-021-01893-7 (PMC9849195; doi:10.1007/s00248-021-01893-7)
Supplement: Supplementary file 1 — Supplementary file1 (PDF 2654 KB) [file 248_2021_1893_MOESM1_ESM.pdf]

Supplementary Information (SI): Intercomparison of two fluorescent dyes to visualize parasitic fungi (Chytridiomycota) on phytoplankton

Isabell Klawonn, Susanne Dunker, Maiko Kagami, Hans-Peter Grossart, and, Silke Van den Wyngaert

Material and methods

Text S1. Chemicals

Dye solutions

Calcofluor White (CFW, Fluorescent Brightener 28, Merck 910090)

- Stock solution (1g L^{-1}):
  - dissolve 100 mg CFW powder in 100 mL of deionized water
  - filter solution through a 0.2 µm membrane filter (PES)
  - storage at room temperature (RT) in the dark

Wheat Germ Agglutinin (WGA, Alexa Fluor™ 488 Conjugate, ThermoFisher W11261)

- Stock solution (1 g L^{-1}):
  - add 5 mL deionized water to 5 mg WGA-Alexa Fluor™ 488 Conjugate powder
  - aliquot into ca. 100–500 µL, to avoid repeated freeze-thawing of the stock solution
  - storage at -20°C (according to the manufacturer instruction, the stock solution is stable for one year)

Fixative solutions

Alkaline (basic) Lugol’s solution

- 10 g Potassium iodide (KI)
- 70 mL dH2O
- 5 g Iodine (I_2)
- 3 g Sodium acetate (CH_3COONa)
- Storage at RT or 4°C in an amber glass bottle

Acidic Lugol´s solution

- 20 g Potassium iodide (KI)
- 200 mL dH2O
- 10 g Iodine (I_2)
- 20 mL glacial acetic acid
- Storage at RT or 4°C in an amber glass bottle

Neutral Lugol’s solution

- purchased at Sigma-Aldrich (Merck) 62650-100ML-F

Paraformaldehyde (PFA) 10%

- the solution should be prepared under a fume hood
- add 100 mL of dH2O to a glass beaker
- stir and heat to 60°C, while stirring, add 10 g of paraformaldehyde powder
- cover the beaker with parafilm and maintain at 60°C until PFA powder is dissolved (optional step)
- raise the pH for better dissolution by adding 2N NaOH (dropwise, ca. 1 drop per 100 mL)
- after the addition of NaOH, the solution should clear within a couple of minutes (some fine particles may remain)
- do not heat above 70°C
- filter the solution through 0.45 µm membrane filter to remove any remaining particles, and aliquot into ca. 10 mL and store at -20°C

Other chemicals

Sodium thiosulfate anhydrous ($Na_2S_2O_3$, Merck 1.06512, solution for de-staining from Lugol)

- Stock solution (30 g L^{-1})
    - dissolve 1.5 g $Na_2S_2O_3$ in 50 mL of deionized water
    - filter solution through a 0.2 µm membrane filter
    - storage at RT for months
- 20–40 µL of $Na_2S_2O_3$ clears 1 mL Lugol-preserved sample (10 µL Lugol mL^{-1})

Culture medium (modified CHU-10)

- add chemicals as listed in Table S1, except for vitamins and Fe-EDTA
- adjust to pH 6.4 with HCl solution or NaOH solution
- autoclave (121°C, 30 min)
- add vitamins and Fe-EDTA after autoclaving and cooling down to avoid heat damage of vitamins
- storage:
    - CHU-10 medium can be stored at RT for several months
    - CHU-10 medium + vitamins/Fe-EDTA can be stored at RT and should preferably be used within 1–2 months

Table S1. Recipe CHU-10 medium.

Supplement	Stock Solution* g L^{-1}	mL L^{-1}	µmol L^{-1} in the final medium
Na_2SiO_3 ($5H_2O$)	5.8	10	273
$Ca(NO_3)_2$ ($4H_2O$)	57.56	1	244
K_2HPO_4	10	1	57
$MgSO_4$ ($7H_2O$)	25	1	101
Na_2CO_3	20	1	189
add after autoclaving			
Fe-EDTA†	37	1	
F/2 Vitamins‡	see below	1	

*stock solutions stored at RT
†filtered through a 0.2 µm membrane filter (stored at 4°C)
‡sterile-filtered F/2 vitamins (stored at 4°C)

F/2 Vitamins

Supplement	Stock Solution mg mL^{-1}	/100ml dist. H_2O
Vitamin B12	5 mg 5 mL^{-1} distilled H2O	0.1 ml
Biotin	1 mg 10 mL^{-1} distilled H2O	1.0 ml
Thiamine HCl		20 mg

Text S2. Protocol for WGA-CFW dual staining, combined with Utermöhl chambers and fluorescence microscopy

Materials and Reagents

- 2 mL microcentrifuge tubes
- 50 mL centrifuge tubes / measuring cylinder
- Utermöhl chamber (Hydro-Bios No. 435 025-002, Germany, with glass cover plates)
- ddH_2O
- neutral/alkaline Lugol´s solution
- WGA-488 stock solution (1 g L^{-1})
- Calcofluor White stock solution (1 g L^{-1})
- Sodium thiosulfate ($Na_2S_2O_3$, 30 g L^{-1})

Equipment

- Inverted epifluorescence microscope (a Nikon Eclipse Ti2-U, Nikon, Japan was used in this study), equipped with an Utermöhl chamber holder

Procedure

1. Sample fixation

- fix water sample with Lugol´s solution (e.g., final conc. 10 µl mL^{-1})
- we recommend the use of neutral or alkaline Lugol´s solution as acidified Lugol´s solution reduces the pH of the sample outside the recommended pH range of 6.5–8.5 given for Alexa Fluor® dyes. Although Alexa Fluor® dyes are pH insensitive across the range of 4–10 [1], we observed a different WGA-Alexa Fluor™ 488 staining behavior for acidic and neutral/alkaline Lugol-preserved samples (Supplementary Figure S5). The pH in the preserved samples was 4 (acidic Lugol) and ca. 7 (neutral/alkaline Lugol).
- store samples overnight at RT in the dark or 4°C for long-term storage
- we recommend an overnight fixation of samples before staining, to ensure an effective WGA binding

Optional: Before staining, fixed phytoplankton cells can be concentrated by sedimentation, as follows.

- homogenize the sample by gentle shaking, and transfer the desired volume to a measuring cylinder or equivalent (e.g., 50 mL centrifugation tube)
- let the sample undisturbed for at least 24 h at RT, to allow the cells to settle to the bottom
- carefully siphon off 90% of the top water layer (any turbulence and cell re-suspension should be avoided), to reach a 10x enrichment factor of cells in the sample

2. WGA and CFW dual-staining

- transfer 1–2 mL of the sample into a 2 mL microcentrifuge tube
- add 20–40 µl of $Na_2S_2O_3$ (30 g L^{-1} stock solution) and gently shake manually to “clear” the Lugol (sample should become transparent)
- add WGA-Alexa Fluor™ 488 Conjugate and CFW (final concentration of both stains 5 µg mL^{-1}) and mix gently. The order of stain addition does not influence the staining results.
- incubate for 15 min at RT in the dark
- transfer the sample into an Utermöhl chamber and wait ~15 minutes to let the cells sink to the bottom of the chamber (keep chambers in the dark to avoid light exposure).
- the prepared sample can be stored in the fridge for 1–2 days if microscopy is not done immediately

Optional: Storage and staining on filters

- preserve cells with PFA (final conc. 1.5%, fixation overnight at 4°C)
- if cells are analyzed immediately, staining can be done in liquid:
  - add WGA-Alexa Fluor™ 488 Conjugate and CFW (final conc. of both stains 5 µg mL^{-1}) and mix gently
  - incubate for 15 min at RT in the dark
  - filter PFA-preserved cells onto PC filters (0.2 µm, 25 mm used herein)
- if cells are stored at -20°C, cells can be filtered before storage and stained after storage:
  - for staining, the filters are submerged in the dye solution (CFW and WGA, final conc. of both stains 5 µg mL^{-1}) for 15 min in darkness, washed twice with 1 mL medium and 1 mL MilliQ to remove excess dye, followed by air-drying

3. Microscopy

Microscope and filter sets

Microscopy analysis of dual-stained WGA-CFW samples can be performed on an inverted epifluorescence microscope equipped with ultraviolet (for CFW) and blue (for WGA-488) excitation filters (Table S2). Alternatively, WGA-fluorochrome conjugates with different excitation wavelengths can be purchased to match the available microscope filter sets.

UV excitation/long pass emission filters enable simultaneous visualization of CFW fluorescence and phytoplankton autofluorescence and can be combined with transmitted light observation (bright-field or DIC [differential inference contrast]). Likewise, blue excitation/long pass emission filters can be used for this purpose in combination with WGA-488. Such a set-up is convenient for identifying and enumerating phytoplankton species (transmitted light/autofluorescence) and attached chytrid sporangia (CFW/WGA) simultaneously within the same field of view, without the need to switch between fluorescent channels and transmitted light.

Table S2. Filter sets used for epifluorescence microscopy.

Dye	Excitation max. (nm)	Emission max. (nm)	Common optical filters	Specifications of filters used in this study
WGA-488	459 (blue)	519 (green)	FITC, GFP, Alexa fluor 488, Bodipy, 5-FAM, and Fluo-4	FITC-3540C (Ex 482/35; DM 506; BA 536/40, bandpass)
CFW	350 (UV)	432 (blue)	DAPI, Hoechst, Alexa Fluor 350, and Calcofluor-White	Calcofluor White HC BrightLine Basic (Ex 387/11; DM 409; BA 442/46, bandpass)<br><br>DAPI-50LP-A (EX 377/50; DM 409; BA 415 LP, longpass)

Objective lenses
Chytrid infections and sporangia associated with microphytoplankton (20–200 µm) are preferably evaluated with an objective lens of 20X/1.5 or 40X (300X or 400X magnification, respectively). Evaluation of chytrids associated with nanophytoplankton (2–20 µm) is preferably done with an objective lens of 60X-60X/1.5 (600X or 900X magnification, respectively).
Cell counting

Cell counting
- a minimum of 400 individuals of each phytoplankton species (including infected and noninfected cells) should be counted, if possible, to reach a counting precision of 10% within 95% confidence limits [2]
- Guidelines for counting phytoplankton in Utermöhl chambers are detailed in the HELCOM phytoplankton monitoring guide [3]
- The following parameters can be obtained from cell counting
  - abundance of uninfected and infected phytoplankton cells and chytrid sporangia
  - infection prevalence P (%), i.e., the proportion of individuals in the phytoplankton community carrying one or more sporangia: P (%) = [(N_i /N_t) x 100], where N_i is the number of infected phytoplankton cells, and N_t is the total number of phytoplankton cells. Note that phytoplankton cells carrying empty sporangia are often excluded from the calculation of P (%), as they do not contribute further to the population growth of the host or the parasite [4].
  - infection severity, i.e., the number of associated chytrid sporangia per host cell (multiple infections per host cell are possible)

Criteria to identify parasitic chytrids (sporangia) on phytoplankton
- positive CFW and WGA staining of the chytrid structure, or positive staining with at least one of the stains
- stained sporangia have a distinctly outlined globose to ovoid shape and are physically attached to a phytoplankton cell (via stalks and/or rhizoids)
- phytoplankton cell carrying the stained sporangia shows signs of chloroplast degradation, indicated by low red autofluorescence
- stained sporangia display very little or rather no Chl *a* autofluorescence

Text S3. Culturing the model pathosystems

The nine taxonomically different host–chytrid pathosystems (Table 1 in the main text) were grown as batch cultures in CHU-10 medium in 100 mL Erlenmeyer flasks at constant temperature (17°C). The light–dark cycle was 16:8 h, providing 40 µmol photons m^{-2} s^{-1} during the 16-h light phase. The pathosystems were maintained by transferring 0.5–1 ml of the infected culture (infection prevalence 50–95%) to 60 ml of non-infected host cells at 7–14 d intervals [5, 6]. The co-cultures were not exposed to continuous shaking, but cells were gently resuspended through manual shaking at least once per week.

Table S3. Phytoplankton host and chytrid strains used in this study with available Genbank accession numbers for rbcL (ribulose-1,5-bisphosphate carboxylase) and rRNA genes (18S/28S - small/large subunits of eukaryotic ribosomal RNA, ITS - Internal transcribed spacer).

			**Genbank accession no.**			
Species	**Host/chytrid**	**Strain**	**rbcl**	**18S**	**ITS**	**28S**
Ulnaria sp.	host	HS-SYN2	LC483762	-	-	-
Staurastrum sp.	host	STAU1	-	-	KY555736	-
Eudorina elegans	host	PAN1	MG670588	-	MG597011	-
Yamagishiella unicocca	host	PAN4	MG655265	-	MG597012	-
Zygophlyctis planktonica	chytrid	SVdW-SYN-CHY1	-	LC482219	LC482220	LC482221
Staurastromyces oculus	chytrid	STAU-CHY3	-	KY350147	KY350146	KY350145
Endocoenobium eudorinae	chytrid	SVdW-EUD1	-	MG605053	-	MG605050
Dangeardia mamillata	chytrid	SVdW-EUD2	-	MG605054	-	MG605051
Algomyces stechlinensis	chytrid	SVdW-EUD3	-	MG605055	-	MG605052

Text S4. Statistical analysis

Statistical differences between two (non-paired) samples were calculated using the Mann–Whitney test for non-normally distributed data, the Welsh-test for normally distributed data with non-equal variance, and the t-test for normally distributed data with equal variance. Normal distribution was verified using the Shapiro-test and data variance with the F-test. Statistical differences between multiple groups were determined with the Kruskal–Wallis test (if the non-normal distribution was given, with Bonferroni correction for *p*-value adjustment, also called Kruskal–Wallis H-test or one-way ANOVA on ranks). Data were considered statistically significant when the *p*-value was <0.05. Data were processed and plotted in RStudio (3.6.1) and Origin 2021.

Results and discussion

Additional figures (S1–S5)

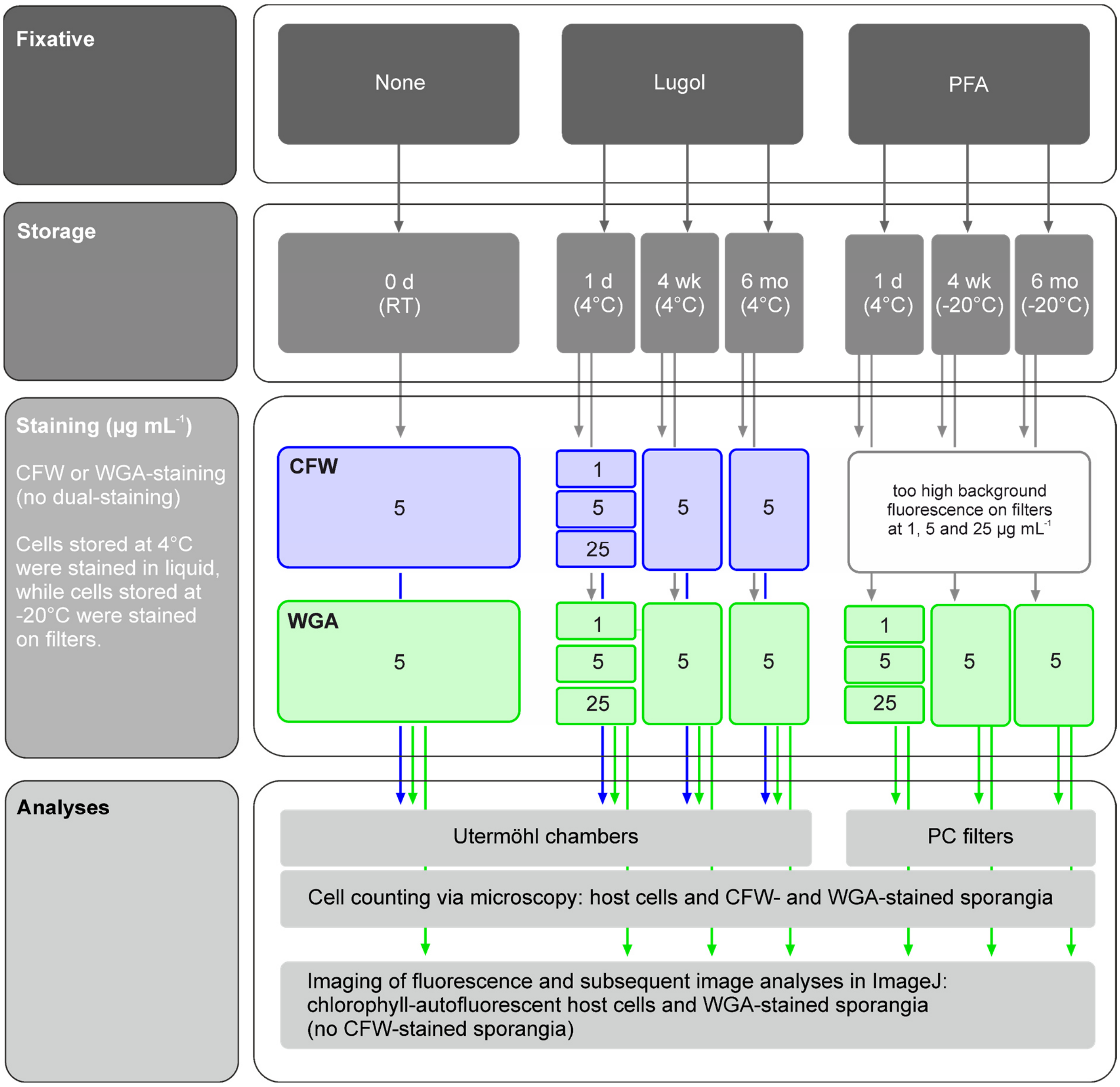

Figure S1. Workflow for testing various fixatives, storage times, dye concentrations, and sample preparation types, using the *Asterionella–Rhizophydiales* #1 co-culture. The arrows indicate the step-wise workflow. Samples were left without fixative, or preserved with Lugol or PFA. Non-fixed, live samples were stained and counted immediately (0 days), while Lugol-/PFA-preserved samples were analyzed after 1 day (1 d), 4 weeks (4 wk), or 6 months (6 mo). Samples that were stored for 1 d were stained with three different dye concentrations (1, 5, and 25 µg mL^{-1}), to test the effect of different dye concentrations on the staining effectivity. Samples that were stored for either 1 d, 4 wk or 6 mo were stained with 5 µg mL^{-1}, to test the effect of different storage times. Lugol-preserved cells (chytrid sporangia) were counted in Utermoehl chambers, while PFA-preserved samples were counted on PC filters. The fluorescence intensity was analyzed for chlorophyll-autofluorescent host cells and WGA-stained sporangia, but not for CFW-stained sporangia.

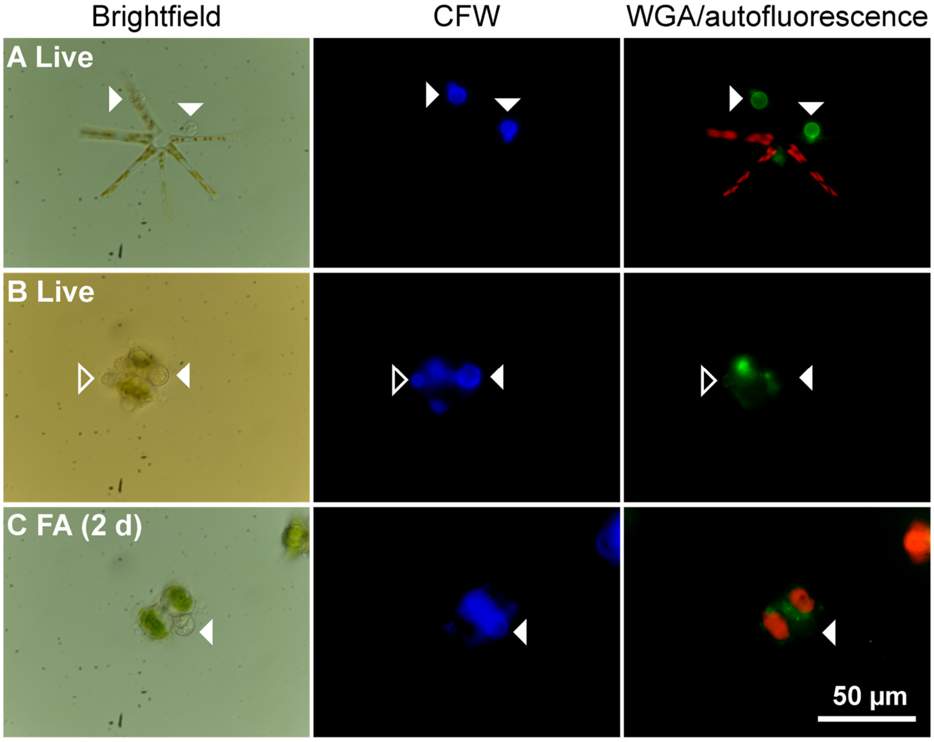

Figure S2. Micrographs depicting the CFW and WGA staining pattern in the **A** *Asterionella–Rhizophydiales* #1 and **B/C** *Staurastrum– Staurastromyces* pathosystems in live (no preservation) and FA-preserved samples (2 days fixation). In the *Asterionella–Rhizophydiales* sp. #1 system, CFW and WGA visualized mature sporangia even when no fixative was added (**A**, live). By contrast, in the *Staurastrum–Staurastromyces* system, encysted zoospores (open arrowheads) and mature sporangia (filled arrowheads) were not fully stained with WGA in live samples and even after 2 days of FA fixation, whereas CFW stained mature sporangia in both live and FA-preserved samples **(B/C)**. In contrast to all other microscopy images shown in this manuscript, these images were taken under a Zeiss LSM710 (Zeiss, Germany, CFW: Fs49 Ex G 365 nm / Em BP 445/50 nm, WGA: Fs38 Ex BP 470/40 nm / Em BP 525/50 (bandpass filter, only WGA signal emitted with WGA excitation, **B**) or Fs9 Ex BP 450–490 nm / Em LP 515 nm (longpass filter, also chlorophyll autofluorescence emitted with WGA excitation, **A and C**), camera: Raspberry Pi HQ camera V1.0 2018, Cambridge, UK).

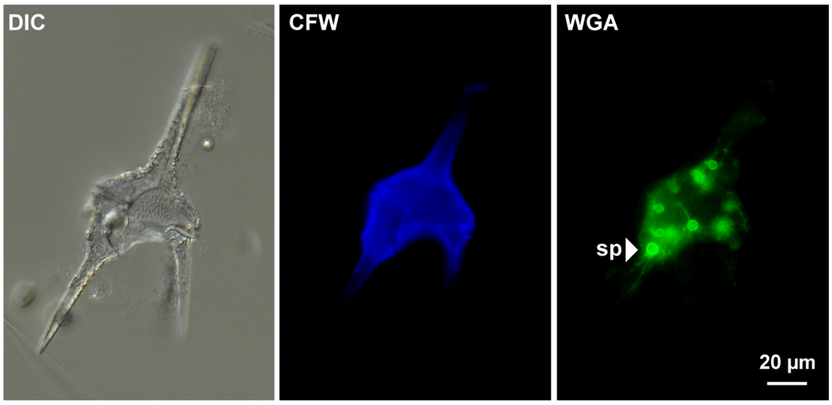

Figure S3. Micrographs of *Ceratium* (dinoflagellate) with associated chytrids. Chytrid sporangia were not visible after CFW-staining (but cellulosic cell walls of *Ceratium* were CFW-stained), while WGA effectively visualized associated sporangia (sp) and rhizoids. DIC - differential interference contrast.

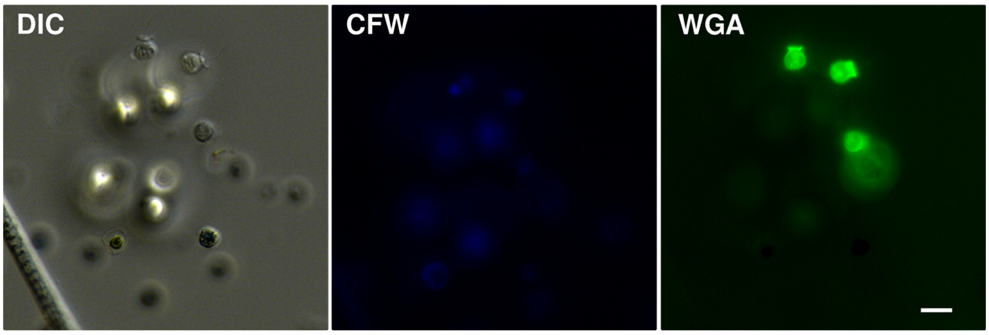

Figure S4. Micrographs of a colonial green alga with attached choanoflagellates. Choanoflagellates were stained with WGA, illustrating an example of a “false positive” detection with WGA. The scale bar is 10 µm. DIC - differential interference contrast

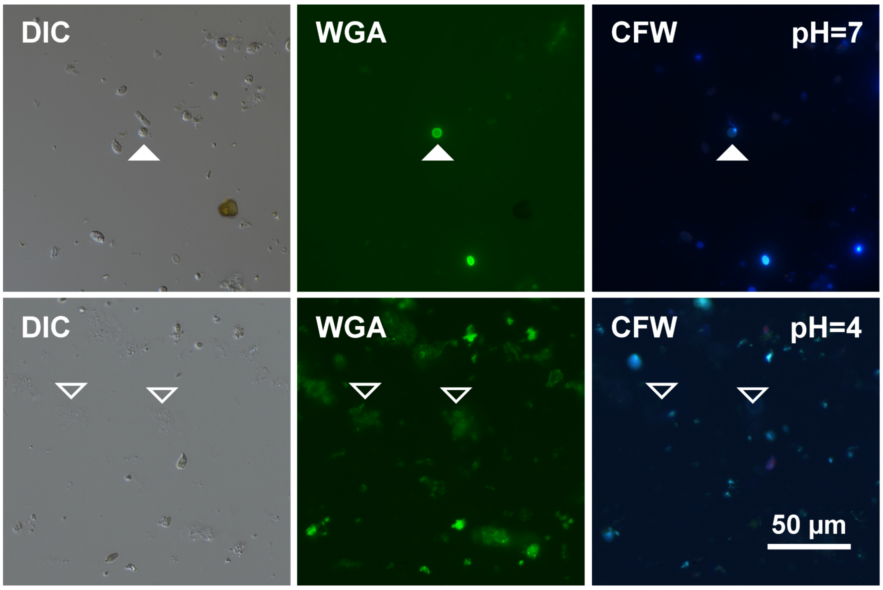

Figure S5. WGA and CFW staining of Baltic Sea water after cell preservation with neutral (pH=7, **upper panel**) and acidic Lugol (pH=4, **lower panel**). At low pH, WGA bond to flocculated particles (open arrowhead), which were not visible in the samples at neutral pH. The filled arrowhead in the upper panel points to a chytrid sporangium. DIC - differential interference contrast.

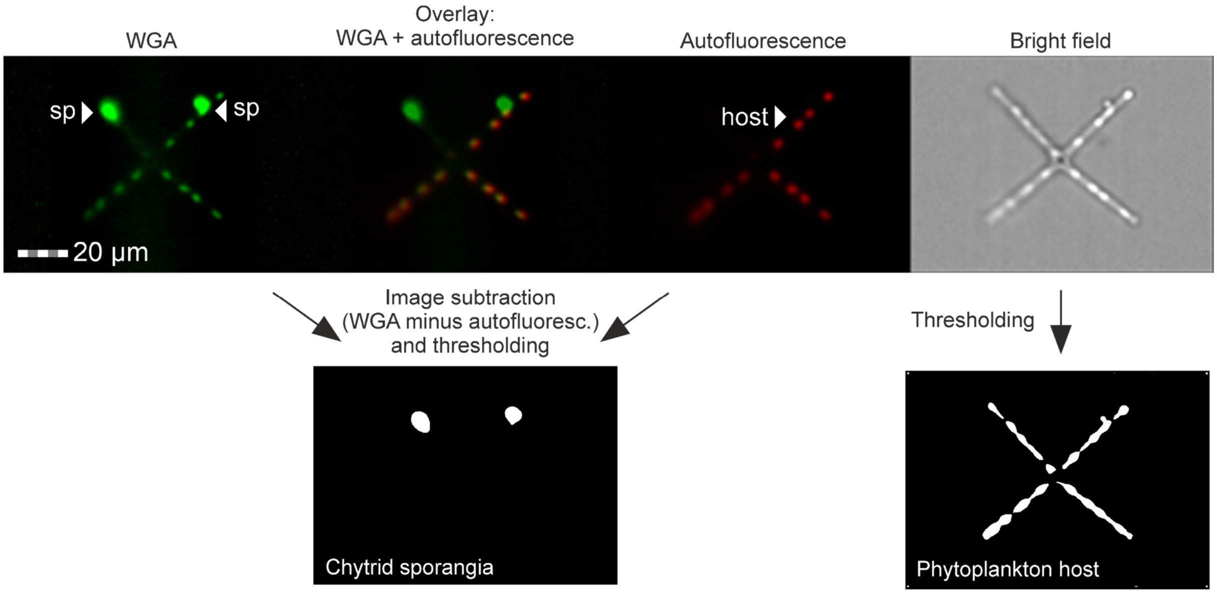

Figure S6. Image output from flow cytometry analyses of the *Asterionella–Rhizophydiales* pathosystem (WGA-stained). Images display two mature sporangia (sp) and four host cells (host). Both cell types could be separated through image analyses in ImageJ, to allow for automatized enumeration of parasite and host cell abundances. The WGA-stained sporangia could be separated after subtracting the host's autofluorescence. For the enumeration of host cells, the bright field image was best applicable.

References

1. N. Panchuk-Voloshina *et al.* (1999) Alexa Dyes, a Series of New Fluorescent Dyes that Yield Exceptionally Bright, Photostable Conjugates. *Journal of Histochemistry & Cytochemistry* **47:**1179-1188.
2. J. W. G. Lund, C. Kipling, E. D. Le Cren (1958) The inverted microscope method of estimating algal numbers and the statistical basis of estimations by counting. *Hydrobiologia* **11:**143-170.
3. HELCOM, Monitoring of phytoplankton species composition, abundance and biomass. https://helcom.fi/wp-content/uploads/2020/01/HELCOM-Guidelines-for-monitoring-of-phytoplankton-species-composition-abundance-and-biomass.pdf. (accessed 31/08/2021).
4. A. S. Gsell, L. N. de Senerpont Domis, E. van Donk, B. W. Ibelings (2013) Temperature alters host genotype-specific susceptibility to chytrid infection. *PLoS ONE* **8:**
5. S. Van den Wyngaert, K. Rojas-Jimenez, K. Seto, M. Kagami, H. P. Grossart (2018) Diversity and hidden host specificity of chytrids infecting colonial volvocacean algae. *J. Eukaryot. Microbiol.* **65:**870-881.
6. S. Van den Wyngaert, K. Seto, K. Rojas-Jimenez, M. Kagami, H. P. Grossart (2017) A new parasitic chytrid, *Staurastromyces oculus* (Rhizophydiales, Staurastromycetaceae fam. Nov.), infecting the freshwater desmid *Staurastrum* sp. *Protist* **168:**392-407.
